# The neglect of child neglect: a meta-analytic review of the prevalence of neglect

**DOI:** 10.1007/s00127-012-0549-y

**Published:** 2012-07-15

**Authors:** Marije Stoltenborgh, Marian J. Bakermans-Kranenburg, Marinus H. van IJzendoorn

**Affiliations:** Centre for Child and Family Studies, Leiden University, PO Box 9555, 2300 RB Leiden, The Netherlands

**Keywords:** Physical neglect, Emotional neglect, Meta-analysis, Epidemiology

## Abstract

**Purpose:**

The aim of the current meta-analysis was to provide an estimate of the prevalence of physical and emotional neglect by integrating prevalence figures from the body of research reporting on neglect. An attempt was also made to unravel the substantial variation in prevalence figures reported in primary studies by analyzing the effects of procedural factors and sample characteristics on combined prevalence rates.

**Methods:**

Studies providing prevalence rates of child neglect were searched using electronic databases, exploring specialized journals, and by searching references of publications for other relevant studies. Data were extracted using a coding system. Intercoder reliability was satisfactory. A comprehensive meta-analysis was conducted.

**Results:**

Child physical neglect prevalence rates were found for 13 independent samples with a total of 59,406 participants, and child emotional neglect prevalence rates were found for 16 independent samples with a total of 59,655 participants. The overall estimated prevalence was 163/1,000 for physical neglect, and 184/1,000 for emotional neglect, with no apparent gender differences. The influence of research design factors on the prevalence of physical neglect was more pronounced than on the prevalence of emotional neglect. Studies on physical neglect in ‘low-resource’ countries were conspicuously absent.

**Conclusions:**

Child neglect is a problem of considerable extent, but seems to be a neglected type of maltreatment in scientific research. This is illustrated by the deplorable dearth of studies on child neglect, especially in low-resource countries. Recommendations for the design of future prevalence studies are proposed.

**Electronic supplementary material:**

The online version of this article (doi:10.1007/s00127-012-0549-y) contains supplementary material, which is available to authorized users.

## Introduction

Although the consequences of child neglect seem to be as important as those of the more active types of abuse and neglect is the most frequent category of child maltreatment recorded by child protection agencies [1], child neglect has not been the primary focus of many empirical studies and it is unclear how often child neglect occurs. In the existing literature, prevalence rates of child neglect ranged from 1.4 % [3]^1^ to 80.1 % [4]. This substantial variation underlined the need for a meta-analytic synthesis to provide an overview of child neglect prevalence and to search for determinants of the variation in prevalence estimates. Neglect has been defined by the Consultation on Child Abuse Prevention [5] as

...the failure to provide for the development of the child in all spheres: health, education, emotional development, nutrition, shelter, and safe living conditions, in the context of resources reasonably available to the family or caretakers and causes or has a high probability of causing harm to the child’s health or physical, mental, spiritual, moral or social development. This includes the failure to properly supervise and protect children from harm as much as is feasible. (p. 15)

Different subtypes of neglect exist. *Physical neglect* refers to the failure to meet children’s physical needs, and includes for example the failure to provide adequate nutrition, clothing, personal hygiene, supervision, and medical attention. *Emotional neglect* refers to the failure to meet children’s emotional needs, and includes for example the failure to provide adequate nurturance and affection, allowing children to be witnesses of domestic violence, to knowingly permit maladaptive behavior by the child, the failure to seek care for emotional of behavioral problems, and the failure to provide adequate structure. *Educational neglect* refers to the failure to provide the care and supervision that are necessary to secure a child’s education. It includes for example failing to enroll a child of mandatory school age in school, permitting chronic absence from school, and failing to attend to special educational needs.

The consequences of neglect seem to be as important as those of abuse [1]. The documented short-term effects of childhood neglect encompass increased risk for childhood internalizing and externalizing behavior and a lack of ego resiliency [6], as well as delays in cognitive and emotional development [7]. The reported long-term effects of childhood neglect include substance abuse [8], diminished economic well-being [9], risky sexual behavior [10], an increased risk for posttraumatic stress disorder [11], a non-standard attachment style [12], an increased likelihood of using social services [13], and an increased likelihood to behave violently [14]. To determine the overall prevalence of physical and emotional neglect, we conducted a meta-analysis of the available studies and also examined the influence of sample characteristics and methodological factors on the reported prevalence.

### Measurement of neglect

Variability existed among studies with respect to the number of items used to establish physical or emotional neglect ranging from one (e.g., [15]) to eight items (e.g., [16]). The number of items used might influence the reported prevalence, because multiple items may include more—and more specific—information about neglect than a single item. For example, in a study by Young et al. [17], physical neglect was assessed with a single item in which respondents replied “never true”, “rarely true”, or “sometimes true” to the statement “There was someone to take care of you and protect you.” (p. 1208). This statement is rather general and open to subjective interpretation by the respondents. In a study by Scher et al. [18], physical neglect was measured with the Childhood Trauma Questionnaire [19]. The CTQ contains five physical neglect items such as “I didn’t have enough to eat when I was growing up.”, which respondents had to rate on a five-point scale ranging from “never true” to “very often true”. These items are behaviorally specific and relatively objective, even though there is still some room for personal interpretation. The same variability existed with regard to questions about emotional neglect. An example of a global question open to subjective interpretation is “You felt loved.”, which had to be rated on a three-point scale (never true; rarely true; sometimes true) [17]. Examples of more behaviorally specific questions are “Were you forced to work?” or “Was your birthday always remembered?” [20].

Another issue of interest is whether questionnaires or interviews are used, and not much is known about this possible source of influence on reported neglect prevalence. A clue as to what to expect may come from CSA research, but findings are equivocal. Some reviews have noted that studies using interviews yield higher prevalence rates than those using questionnaires [21, 22], while others have not reported such a difference [23, 24]. In our meta-analysis on the prevalence of CSA, we found similar figures for face-to-face interviews and questionnaires, but somewhat lower prevalences when telephone interviews or computer-based questionnaires were used [2].

Both questionnaires and interviews are based on self-report and on retrospective recollection of events, contrary to reports by informants such as professionals in health care and child protective services that rely on observations and thus do not rely on potentially biased memories of the respondents of self-report studies. A large difference in prevalence has been consistently found in meta-analyses on the prevalence of child sexual, physical, and emotional abuse [2, 25, 26], with informant rates being a fraction of self-reported rates. One of the reasons for this large difference may be that informants may capture only the top of the proverbial iceberg compared to self-report studies. On the other hand, retrospective self-reports may be influenced by current mood and experiences, and the chronicity and context are often not taken into account, which may result in uncertainty about the reported experiences.

### Procedural factors

A procedural factor that varied between individual studies was sample size. Whether sample size influences reported prevalence is not clear, but one might argue that larger samples might better represent the population and as such provide a better and certainly more precise [i.e., with a smaller confidence interval (CI)] estimate of the prevalence of neglect. However, it is unknown whether a better representation of the population is associated with a higher or a lower prevalence of neglect. Sampling procedure was another procedural factor that differed between studies. Various types of convenience samples were often used, such as women recruited on postpartum wards of six hospitals in the Greater Toronto Area [15], members of a health plan in San Diego [28], or undergraduate female Latina psychology students at a private urban university in Texas [27]. Other samples were randomly or modified randomly drawn, as in a national computer-generated stratified random sample in the USA [29] or a New Zealand urban region birth cohort [30]. The influence of sampling method on reported neglect prevalence is unknown. However, convenience samples have been shown to lead to biased results in other areas of investigation [31].

### Sample characteristics

A sample characteristic that might influence the reported prevalence of neglect is socioeconomic status (SES). In individual studies, low SES was often associated with more child neglect. Evidence came from both informant-based studies and studies using self-report measures of neglect (e.g., [32–35]). Gender differences in the prevalence of neglect were not to be expected as a meta-analysis on risk-factors for neglect did not find gender to be a risk factor [35], and the fourth National Incidence Study (NIS-4) [36] did not find gender differences in the prevalence of neglect either.

### This study

The current meta-analysis aimed at providing an estimate of the prevalence of physical and emotional neglect by integrating prevalence figures from the body of research reporting on neglect. We attempted to unravel the substantial variation in prevalence figures reported in primary studies by analyzing the effects of procedural factors and sample characteristics on combined prevalence rates. We expected combined rates to be similar for women and men and higher in studies with low SES samples. With respect to the other procedural factors and sample characteristics analyzed, the analyses were exploratory due to the absence of firm evidence that could be derived from existing literature.

## Method

### Literature search

Three search methods were used to identify eligible studies published between January 1980 and January 2008. First, we searched the electronic databases PubMed, Online Contents, Picarta, ERIC, PsychINFO, and Web of Science for empirical articles using the terms *prevalence* and/or *incidence* combined with one of the following terms: (child*) (*physical/emotional/educational*) *neglect*. Studies that were found with the search terms (child*) (*sexual/physical/emotional*) *maltreatment*, (*sexual/physical/emotional*) *abuse*, and *victimization* were also included when the prevalence of physical, emotional, or educational neglect was reported. Second, we electronically searched the specialized journals *Child Abuse & Neglect* and *Child Maltreatment* with the same terms as mentioned above. Third, the references of the collected papers, dissertations, and book chapters were searched for relevant studies, as were other reviews and meta-analyses on childhood neglect. The abstracts of the retrieved studies were screened for eligibility of participation in the meta-analysis. Studies were included if the prevalence of at least one of the types of neglect was reported (a) in terms of proportions at child level (excluding studies only reporting estimates of the family level), (b) for victims under the age of 18 years in (c) non-clinical samples, and (d) if sufficient data were provided to determine this proportion as well as the sample size.

If publications reported on the same sample or on overlapping samples, the publication providing the maximum information was included in the meta-analysis. Thus, the independence of samples and the inclusion of every participant only once in the pertinent meta-analyses were ascertained. When a publication reported the prevalence of neglect for more than one sample separately, for example for male and female participants or for participants of different ethnicities, these sub-samples were treated as independent studies.

### Data extraction

We coded two types of moderators: sample characteristics and procedural moderators (see Supplemental Appendix A for coding system). *Sample characteristics* comprised the gender distribution in the sample (100 % female, 100 % male, or mixed), the geographical area from which the sample originated (Australia/New Zealand, North America, Europe, Africa, South America, Asia), the level of economic development of the sample’s country of origin according to the World Economic Outlook Database [37] (high-resource vs. low-resource), the predominant ethnicity of the sample for studies originating from the USA and Canada (African American, Asian, Caucasian, or Hispanic), the predominant SES of the sample (high, moderate, or low), the age of the respondent at the time of assessment, and whether the respondent were adults retrospectively reporting on their childhood experiences or children at the time of assessment.

*Procedural moderators* included the following variables: the type of instrument used (questionnaire or interview), whether the instrument used was validated (yes or no), the number of questions asked (recoded into two categories: up to two questions vs. three or more questions), in case of emotional neglect, whether it was based on witnessing domestic violence only or on more indicators, the sampling procedure (randomized or convenience), the response rate [low (<80.0 %) vs. high (≥80.0 %)], and the sample size [small to moderate (<1,000) vs. large (≥1,000)]. Agreement between the two coders for moderators and outcome variables was satisfactory (mean kappa for categorical variables 0.89, percentage agreement on average 93 %, mean intraclass correlations for continuous variables 0.93).

### Meta-analytic procedures

Meta-analysis was performed using the comprehensive meta-analysis (CMA) program [38]. For each study, the proportion of neglected children was transformed into a logit event rate effect size and the corresponding standard error was calculated [39]. After the analyses, the logits were retransformed into proportions to facilitate interpretation of the results. The coded outcome was the proportion of children physically or emotionally neglected. No outlying effect sizes were detected on the basis of standardized *z* effect-size values larger than 3.29 or smaller than −3.29, thus belonging to the extreme 1 % of a normal distribution. Combined effect sizes were computed using CMA.

Significance tests and moderator analyses were performed through random effects models [40]. Fixed effects models are based on the assumption that effect sizes observed in studies estimate the corresponding population effect with random error that stems only from the chance factors associated with subject-level sampling error in that study [39, 41]. This assumption is not made in random effects models [42]. Random effects models allow for the possibility that there are also random differences between studies that are associated with variations in procedures, measures, or settings that go beyond subject-level sampling error and thus point to different study populations [39]. To test the homogeneity of the overall set and specific sets of effect sizes, we computed *Q* statistics [38]. In addition, we computed 95 % CIs, again based on random estimates, around the point estimate of each set of effect sizes. *Q* statistics and *p* values were also computed to assess differences between combined effect sizes for specific subsets of studies grouped by moderators. Again, the more conservative random effects model tests were used. Contrasts were only tested when at least two of the subsets consisted of at least four studies [43]. We conducted all moderator analyses with the original sample sizes and with a winsorized sample size [44] for the large study of Young et al. [17] that had an outlying value on sample size, reducing the original sample size of 41,482 to 11,000. The results were similar. Therefore, the results of the analyses with the original sample size are reported.

Some publications reported prevalences of physical and emotional neglect for the same samples, resulting in a partial overlap of the sets of studies. It was therefore impossible to make a direct comparison between the combined prevalence rates of the complete sets of physical and emotional neglect studies. Instead, we used 85 % CIs as a conservative way of testing [45] whether the prevalences of physical and emotional neglect were statistically significantly different. Non-overlapping 85 % CIs suggest a significant difference between combined effect sizes [46, 47]. For continuous moderators, Fisher’s *z* scores were used in weighted least squares meta-regression analyses.

We used the “trim and fill” method [48, 49] to calculate the effect of potential publication bias on the outcomes of the meta-analyses. Using this method, a funnel plot is constructed of each study’s effect size against its precision (usually plotted as 1/SE). These plots should be shaped like a funnel if no publication bias is present. However, since smaller studies and studies with non-significant results are less likely to be published, studies in the bottom left-hand corner are often omitted [49, 50]. The *k* left-most studies considered to be symmetrically unmatched are trimmed and their missing counterparts imputed or “filled” as mirror images of the trimmed outcomes. This then allows for the computation of adjusted overall effect sizes and CIs [50, 51]. We also examined the stability of the results using the ‘jackknife’ procedure, analyzing whether the overall effect size changed significantly when the combined effect sizes were calculated after the successive removal of one effect size [38]. We calculated the fail-safe number, being the number of studies with average sample sizes and null outcomes that would be required to bring the combined effect size of the meta-analysis to a non-significant level [52]. Rosenthal [41] suggested that 5*k* + 10, where *k* is the number of studies included, may be considered a general criterion for robustness.

## Results

The search procedure described above yielded 16 publications (see reference list and Supplemental Appendix B) covering reports on the self-reported prevalence of physical neglect (13 samples; 59,406 participants) and emotional neglect (16 samples; 59,655 participants). We also found four publications in which informant reports were used for the prevalence of physical neglect (2 samples), emotional neglect (1 sample), and educational neglect (1 sample). These studies were not included in the current meta-analysis as the number of studies was too small to warrant further analyses. The distribution among the categories of the moderators within the sets of physical and emotional abuse studies can be found in Table 1. Supplemental Appendix C provides an overview of the characteristics of the studies included in both sets.

**Table 1:** Results of moderator analyses for self-report studies: number of studies and participants, and combined prevalence including 95 % confidence intervals (CI)

	Physical neglect					Emotional neglect				
	*k* ^c^	*N*	Combined prevalence (%)	95 % CI	Contrast *Q* ^a^	*k* ^c^	*N*	Combined prevalence (%)	95 % CI	Contrast *Q* ^a^
Overall estimate	13	59,406	16.3**	12.1–21.5	1,122.82^d, **^	16	59,655	18.4**	13.0–25.4	2,554.35^d, **^
Sample characteristics										
Gender					0.07					0.36
Female	6	4,115	15.2**	6.9–30.3		9	11,079	16.1**	10.6–23.6	
Male	6	44,463	17.5**	8.1–34.0		5	46,831	13.6**	7.7–22.9	
Mixed	1	10,828	11.7	1.5–53.1		2	1,745	53.8	30.3–75.7	
Continent					N/A					N/A
Asia						3	1,583	30.1**	19.8–42.9	
Australia						2	1,019	40.0	26.3–55.5	
Europe	2	2,869	6.5**	3.0–13.7						
South America										
USA/Canada	11	56,537	19.2**	14.2–27.1		11	57,053	14.5**	11.4–18.3	
Economic development					N/A					N/A
High resource	13	59,406				13	58,072	17.2**	13.2–22.1	
Low resource						3	1,583	28.5**	16.9–43.7	
Ethnicity^b^					N/A					N/A
African American	1	178	30.0	8.4–66.7		1	178	33.0*	21.0–47.6	
Asian	2	470	55.1	29.3–78.4						
Caucasian	7	45,061	12.5**	7.4–20.4		7	55,941	12.2**	10.1–14.8	
Hispanic						1	112	33.9*	21.0–49.7	
SES					N/A					N/A
Predominantly low	1	178	30.0	9.2–64.5		3	2,040	46.3	30.6–62.7	
Predominantly moderate	7	43,501	32.2**	21.7–44.9		8	52,726	11.7**	8.0–16.8	
Predominantly high	2	2,030	1.4**	0.5–4.1		1	3,527	14.4**	5.1–34.5	
Respondent					N/A					N/A
Adult	11	57,376	22.7**	17.2–29.2		13	57,681	14.4**	10.8–19.0	
Child	2	2,030	1.4**	0.6–3.4		3	1,974	46.8	30.8–63.4	
Procedural moderators										
Witnessing domestic violence^e^										0.18
EN						6	44,067	16.0**	7.3–31.8	
EN = witnessing only						10	15,588	19.6**	10.9–32.6	
Type of instrument					N/A					10.94**
Interview	3	2,208	4.6**	2.2–9.5		4	4,724	30.4	15.4–51.2	
Questionnaire	8	56,231	23.2**	16.2–32.1		9	53,764	21.3**	13.1–32.7	
Instrument validated					50.01**					0.67
No	6	57,209	5.7**	3.7–8.6		12	58,398	20.0**	13.3–28.8	
Yes	7	2,197	34.7**	26.1–44.3		4	1,257	14.2**	6.7–27.8	
Number of questions					19.19**					2.10
1 or 2	4	54,340	5.1**	2.7–9.4		7	55,796	14.1**	9.5–20.5	
3 or more	8	4,888	24.6**	17.1–34.1		7	3,569	21.0**	14.4–29.5	
Sampling procedure					22.55**					2.42
Convenience	6	42,712	37.9	24.1–53.9		9	52,397	14.6**	9.1–22.5	
Randomized	7	16,694	6.5**	3.7–11.4		7	7,258	24.3**	15.1–36.9	
Response rate					61.18**					35.90**
Low to moderate	8	58,176	7.8**	5.7–10.7		9	56,253	12.1**	8.9–16.3	
High	5	1,230	43.3	33.2–54.0		5	3,059	43.8	33.2–54.9	
Sample size					50.01**					0.79
Small to moderate	7	2,197	34.7**	26.1–44.3		11	3,946	16.5**	10.6–24.8	
Large	6	57,209	5.7**	3.7–8.6		5	55,709	22.9**	12.5–38.2	

*EN* emotional neglect

* p < 0.05, ** *p* < 0.01

^a^Subgroups with *k* < 4 or ‘other’ categories are excluded from contrasts [43]

^b^For the subset of studies originating from the USA and Canada

^c^Differences in totals of *k* are due to the exclusion from the pertinent analysis of studies with missing values

^d^ *Q* for heterogeneity of the set of studies

^e^EN: studies in which emotional neglect was based on several indicators; EN = witnessing only: studies in which emotional neglect was based on witnessing domestic violence only

### Combined prevalence

**Fig. 1:**
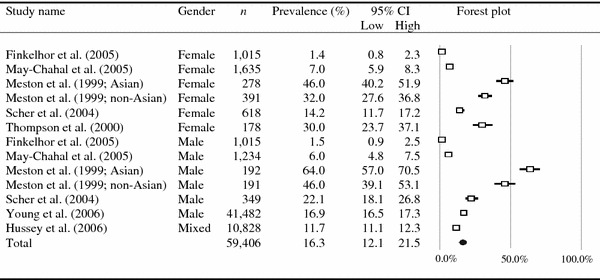
Statistics and forest plot for studies participating in the meta-analysis of physical neglect

**Fig. 2:**
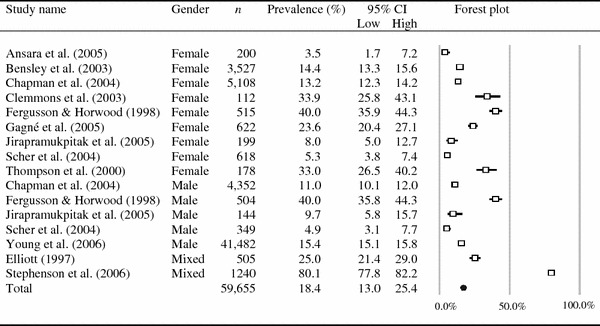
Statistics and forest plot for studies participating in the meta-analysis of emotional neglect

The combined self-reported prevalence for the set of physical neglect studies was 16.3 % (*k* = 13, *N* = 59,406; 95 % CI 12.1–21.5; *p* < 0.01), and the combined self-reported prevalence for emotional neglect was 18.4 % (*k* = 16, *N* = 59,655; 95 % CI 13.0–25.4; *p* < 0.01; see Table 1). Figures 1 and 2 show the distribution of the prevalence figures reported by the included studies on physical and emotional neglect, respectively. Both sets of studies were heterogeneous (for statistics, see Table 1). The 85 % CIs of the combined prevalence rates of the complete sets of physical and emotional neglect studies overlapped (13.1–20.0 and 14.3–23.4 %, respectively), indicating that the difference in combined prevalence was not statistically significant.

Duval and Tweedie’s [48, 50] trim and fill method revealed that asymmetrical publication bias was unlikely for both types of neglect. The jackknife procedure yielded the same point estimate and CIs for both types of neglect, which indicated stability of our findings. The fail-safe number—the number of studies with null result needed to cancel out the significance of the combined prevalence—was 4,531 for the set of physical neglect studies and 7,538 for the emotional neglect studies. Thus, 4,531 physical neglect studies and 7,538 emotional neglect studies with null results would be needed to reduce the combined prevalences to non-significance.

The results of the moderator analyses are presented separately for physical and emotional neglect. The subsets of all moderator analyses remained heterogeneous, indicating that the sample characteristics and procedural factors used in this meta-analysis did not fully explain the variation in prevalence rates of physical and emotional neglect.

### Physical neglect

#### Sample characteristics

The result of the moderator analysis for gender was not significant, indicating that physical neglect occurred at approximately the same rate among females and males (see Table 1). Moderator analyses for the other sample characteristics could not be carried out due to the small set of physical neglect studies leading to <4 studies per category.

#### Procedural moderators

The use of validated instruments yielded a significantly higher prevalence for physical neglect than the use of non-validated instruments. The combined prevalence was significantly lower when one or two questions were used to assess the occurrence of physical neglect than when three or more questions were used. A meta-regression using the number of questions as predictor and the logit event rate as dependent variable revealed a significant model with a positive slope, indicating an increase of reported prevalence with an increasing number of questions (*z* = 3.04, *p* = 0.002) and thus confirming the result of the moderator analysis. The combined prevalence of studies using convenience samples was significantly higher than that of studies using randomized samples. The combined prevalence of studies with low or moderate response rates was significantly lower than those with high response rates. For studies with small to moderate sample sizes, a higher combined prevalence was found than for studies with large sample sizes. The contrast between studies using interviews or questionnaires could not be tested due to the small set of physical neglect studies using interviews.

### Emotional neglect

#### Sample characteristics

As for physical neglect, gender was not a significant moderator implying that emotional neglect occurs at about the same rate among females and males (see Table 1). Moderator analyses for the other sample characteristics could not be carried out due to the small set of emotional neglect studies.

#### Procedural moderators

No difference in reported prevalence was found between studies that reported on witnessing domestic violence only and studies that used a more comprehensive definition of emotional neglect. The combined prevalence of studies using interviews was significantly higher than the combined prevalence of studies using questionnaires (see Table 1). The combined prevalence of studies with low or moderate response rates was significantly lower than the combined prevalence of studies with high response rates. The analyses of none of the other procedural moderators reached significance, indicating that no differences existed in the combined prevalence between studies using: validated or non-validated instruments, fewer than three or more than three questions for the assessment of emotional neglect, convenience or randomized samples, and small to moderate or large sample sizes.

## Discussion

Neglect seems to be a neglected type of maltreatment in scientific research. This is apparent from the fact that we could trace only a modest number of studies reporting on the prevalence of neglect: 16 for physical neglect including 59,406 participants, and 13 for emotional neglect including 59,655 participants. These numbers were strikingly low in comparison to a recently published meta-analysis on the prevalence of CSA [2] that yielded over 200 publications using self-report measures of CSA for over 400,000 participants. This illustrates the dearth of studies reporting the prevalence of neglect. Even more telling was the fact that the prevalence of neglect was always reported in combination with reports of the prevalence of CSA, child physical abuse, and/or child emotional abuse, indicating that studies on the prevalence of neglect were by-products rather than a primary interest. Informant studies in which the prevalence of neglect was reported were especially scarce, which precluded us from combining them meta-analytically and from comparing the combined prevalence of studies based on informants and on self-report.

The global prevalence of self-reported child physical neglect was estimated to be 16.3 % or 163 per 1,000 children, and the global prevalence of self-reported child emotional neglect was estimated to be 18.4 % or 184 per 1,000, with no apparent gender differences.

In rather small sets of studies outlying effect sizes and sample sizes may exert a large influence on the estimated effect size. In our set of studies, the largest sample size (*N* = 41,482) was found in the study by Young et al. [17], and the study by Meston et al. [53] reported rather large prevalence rates. However, neither winsorizing the largest sample size nor the jackknife procedure, in which the reported prevalence was calculated when one study at a time is removed, resulted in meaningful changes of the estimated prevalence. Therefore, we can be reasonably certain of the robustness of our meta-analytic results.

Due to the small number of studies, the possible influence of many sample characteristics could not be tested. Also, the distribution of studies among geographical areas of origin of the sample was rather uneven with a large majority of samples originating from North America, no samples from South America, and only few from Asia, Australia, and Europe. The same applied to the level of economic development. All physical neglect samples and a majority of the emotional neglect samples originated from countries that are labeled high resource in the World Economic Outlook Database [37]. This is especially unfortunate because higher prevalence rates of physical neglect may be expected in low-resource countries due to the difficult life circumstances of most parents and children in these countries (as described by, e.g., [54]).

The contrasts based on procedural moderators showed that most procedural factors influenced the prevalence of physical neglect, but not the prevalence of emotional neglect (e.g., the number of questions used to assess neglect, the sampling procedure). Exceptions were whether questionnaires or interviews were used, with questionnaires yielding lower rates of emotional but not of physical neglect, and response rate that showed higher combined prevalences for both types of neglect when studies had high response rates. Differences in moderator effects may be related to differences between physical and emotional neglect. Emotional neglect may be more difficult to rate than physical neglect, as the construct of emotional neglect may be more open to personal interpretation. A rather extreme example of an item that was open to subjectivity was “you felt loved.” to which participants could answer “never true”, “rarely true”, or “sometimes true” [17]. Although one might wonder whether subjectivity can be entirely banned from the measurement of emotional neglect, we recommend the use of multiple, behaviorally specific questions about physical and emotional neglect to rule out at least part of the subjectivity.

We found substantial differences in the prevalence of physical neglect for studies using different types of procedural characteristics. Interestingly, studies with seemingly better procedural characteristics showed on and off higher and lower prevalence rates. For example, randomly drawn samples, preferred from a methodological perspective, showed a lower combined prevalence than convenience samples, but larger numbers of questions yielding more precise information on neglect were associated with a higher combined prevalence, as were higher response rates. In general, conceptual difficulties of defining and measuring neglect are inherent to research on neglect, maybe in particular on emotional neglect which seems the less visible of the two types of neglect. Various studies on neglect used rather different definitions and measurements which might have affected the validity of our meta-analytic findings. The fact that we were unable to find moderators that created homogeneous sub-sets of studies points in the direction of unexplained variations between studies. This is the reason why we used random effect models for our meta-analytic procedures that lead to larger but also more valid confidence boundaries around the point estimates.

Trying to delineate studies with, in order, overall good and suboptimal procedural qualities, two studies are described that might illustrate such procedural differences: May-Chahal and Cawson [16] is an example of a study with better procedural qualities, whereas the Young et al. [17] study seems less optimal. May-Chahal and Cawson [16] reported the prevalence of physical neglect in two randomized samples of 1,634 female and 1,235 male adult participants aged 18–24 years from the UK, with a response rate of 69 %. Eight quite specific items on physical neglect were used, such as “Before you were 12 years old, you always/often went hungry because no one got you meals or there was no food in the house” and “You regularly had to look after yourself because your parents went away”. The physical neglect prevalence was 6.0 % for boys and 7.0 % for girls. As an example of a study with less optimal procedural qualities, Young et al. [17] examined the prevalence of physical neglect in a large convenience sample of 41,482 young male Marine recruits at the Marine Corps Recruit Depot in San Diego, USA, with a response rate 63.6 %. A single item was used to measure physical neglect: “There was someone to take care of you and protect you before the age of 17”, which respondents had to respond to by “never true”, “rarely true”, or “sometimes true”. The physical neglect prevalence was 16.9 % [17]. Interestingly, the physical neglect prevalence reported in the study with the better design features [16] was about half of the prevalence reported in the study with the less optimal procedures [17]. Although no firm conclusion can be drawn from these examples, they might indicate a potential overestimation of the physical neglect prevalence due to less optimal design features of several prevalence studies.

## Conclusion

The current meta-analysis showed that child neglect is a problem of considerable extent, touching the lives of many children. Given the dearth of studies investigating—the prevalence of—child neglect and given the severe consequences of neglect [1], more studies with a primary focus on child neglect should be undertaken. Carrying out studies with a primary focus on child neglect in low-resource countries is especially important, because the body of research in these countries is even more limited than in high-resource countries. Such studies should be methodologically sound, use representative randomized population samples, and should include clear and behaviorally defined operationalizations for physical and emotional neglect. In the current meta-analysis including almost 60,000 participants for each type of neglect, we found a disturbingly high prevalence of physical neglect (163/1,000 cases) and emotional neglect (184/1,000 cases). More than 15 % of the children are estimated to suffer from neglect. Clinical programs to support parents and children at risk for neglect should be made available at a large scale if one wants to reach the millions of families with children suffering from neglect. Although more studies need to be conducted, it is also clear that this high percentage of neglected children is a sufficiently solid evidence base for social policies to make life for these children and their families more bearable, and in accordance with the Universal Children’s Rights [55].

## Electronic supplementary material

Below is the link to the electronic supplementary material.

Supplementary Appendices A–C (PDF 121 kb)

## Footnotes

1 Publications marked with an asterisk are both cited in the text and included in the meta-analysis. A full reference list of the studies participating in the meta-analysis can be found in the Supplemental Appendix.
