# Supplementary material for: The neglect of child neglect: a meta-analytic review of the prevalence of neglect
Source: Soc Psychiatry Psychiatr Epidemiol. 2012 Jul 15;48(3):345–55. doi: 10.1007/s00127-012-0549-y (PMC3568479; doi:10.1007/s00127-012-0549-y)
Supplement: Supplementary file 1 — Supplementary Appendices A–C (PDF 121 kb) [file 127_2012_549_MOESM1_ESM.pdf]

Supplemental Appendix A. *Coding System*

Variable	Coding		Description
Sample characteristics			
Gender distribution in sample	1	Male	
	2	Female	
	3	Mixed	
Continent	1	Australia	Including New Zealand
	2	North America	Including USA and Canada
	3	Europe	
	4	Africa	
	5	South America	
	6	Asia	
Country's level of economic development	1	Low-resource	According to the World Economic Outlook Database [37]
	2	High-resource	
Ethnicity	1	African-American	Predominance in sample, based on reports in the study
	2	Caucasian	
	3	Asian	
	4	Hispanic	
SES	1	Low	Predominance in sample, based on reports in the study
	2	Moderate	
	3	High	
Respondent	1	Child	
	2	Adult	
Procedural moderators			
Evidence maltreatment	1	Informant	
	2	Self-report	
Witnessing domestic violence	1	Several indicators	Coded for emotional neglect
	2	Witnessing domestic violence only	
Type of instrument	1	Interview	
	2	Questionnaire	
Instrument validated	1	No	
	2	Yes	
Number of questions regarding neglect			Continuous; if a range was provided, the minimum number was coded
Sampling procedure	1	Randomized	
	2	Convenience	
Response rate	1	Low to moderate	< 80%
	2	High	≥ 80%
Sample size	1	Small to moderate	< 1,000
	2	Large	≥ 1,000

Supplemental Appendix B. References of publications participating in the meta-analyses

Ansara D, Cohen MM, Gallop R, Kung R, Schei B (2005) Predictors of women's physical health problems after childbirth. J Psychosom Obstet Gynecol 26: 115-125. doi: 10.1080/01443610400023064

Bensley L, Van Eenwyk J, Wynkoop Simmons K (2003) Childhood family violence history and women's risk for intimate partner violence and poor health. Am J Prev Med 25: 38-44. doi: 10.1016/S0749-3797(03)00094-1

Chapman DP, Whitfield CL, Felitti VJ, Dube SR, Edwards VJ, Anda RF (2004) Adverse childhood experiences and the risk of depressive disorders in adulthood. J Affect Disord 82: 217-225. doi: 10.1016/j.jad.2003.12.013

Clemmons JC, DiLillo D, Martinez IG, Degue S, Jeffcott M (2003) Co-occurring forms of child maltreatment and adult adjustment reported by Latina college students. Child Abus Negl 27: 751-767. doi: 10.1016/S0145-2134(03)00112-1

Elliott DM (1997) Traumatic events: Prevalence and delayed recall in the general population. J Consult Clin Psychol 65: 811-820. doi: 10.1037//0022-006X.65.5.811

Fergusson DM, Horwood LJ (1998) Exposure to interparental violence in childhood and psychosocial adjustment in young adulthood. Child Abus Negl 22: 339-357. doi: 10.1016/S0145-2134(98)00004-0

Finkelhor D, Ormrod R, Turner H, Hamby SL (2005) The victimization of children and youth: A comprehensive, national survey. Child Maltreatment 10: 5-25. doi: 10.1177/1077559504271287

Gagné MH, Lavoie F, Hébert M (2005) Victimization during childhood and revictimization in dating relationships in adolescent girls. Child Abus Negl 29: 1155-1172. doi: 10.1016/j.chiabu.2004.11.009

Hussey JM, Chang JJ, Kotch JB (2006) Child maltreatment in the United States: Prevalence, risk factors, and adolescent health consequences. Pediatr 118: 933-942. doi: 10.1542/peds.2005-2452

Jirapramukpitak T, Prince M, Harpham T (2005) The experience of abuse and mental health in the young Thai population. Soc Psychiatr Psychiatr Epidemiol 40: 955-963. doi: 10.1007/s00127-005-0983-1

May-Chahal C, Cawson P (2005) Measuring child maltreatment in the United Kingdom: A study of the prevalence of child abuse and neglect. Child Abus Negl 29: 969-984. doi: 10.1016/j.chiabu.2004.05.009

Meston CM, Heiman JR, Trapnell PD, Carlin AS (1999). Ethnicity, desirable responding, and self-reports of abuse: A comparison of European- and Asian-ancestry undergraduates. J Consult Clin Psychol 67: 139-144. doi: 10.1037//0022-006X.67.1.139

Scher, CD, Forde DR, McQuaid JR, Stein MB (2004) Prevalence and demographic correlates of childhood maltreatment in an adult community sample. Child Abus Negl 28: 167-180. doi: 10.1016/j.chiabu.2003.09.012

Thompson MP, Kaslow NJ, Lane DB, Kingree JB (2000) Childhood maltreatment, PTSD and suicidal behavior among African American females. J Interpers Violence 15: 3-15. doi: 10.1177/088626000015001001

Young SYN, Hansen, CJ, Gibson, RL, Ryan MAK (2006) Risky alcohol use, age at onset of drinking, and adverse childhood experiences in young men entering the US marine corps. Arch Pediatr Adolesc Med 160: 1207-1214. doi: 10.1001/archpedi.160.12.1207

Supplemental Appendix C. *Studies in which the prevalence of physical or emotional neglect was self-reported*

Study	Gender	*N*	Prevalence (%)	Continent	Economic Development[1]	Ethnicity[2, 3] (predominant)	SES[3]	Respondent
Physical neglect								
Finkelhor et al. (2005)	Female	1,015	1.4	North America	High	Caucasian	High	Child
Finkelhor et al. (2005)	Male	1,015	1.5	North America	High	Caucasian	High	Child
Hussey et al. (2006)	Mixed	10,828	11.7	North America	High	--	--	Adult
May-Chahal et al. (2005)	Female	1,635	7.0	Europe	High	--	--	Adult
May-Chahal et al. (2005)	Male	1,234	6.0	Europe	High	--	--	Adult
Meston et al. (1999; Asian)	Female	278	46.0	North America	High	Asian	Moderate	Adult
Meston et al. (1999; non-Asian)	Female	391	32.0	North America	High	Caucasian	Moderate	Adult
Meston et al. (1999; Asian)	Male	192	64.0	North America	High	Asian	Moderate	Adult
Meston et al. (1999; non-Asian)	Male	191	46.0	North America	High	Caucasian	Moderate	Adult
Scher et al. (2004)	Female	618	14.2	North America	High	Caucasian	Moderate	Adult
Scher et al. (2004)	Male	349	22.1	North America	High	Caucasian	Moderate	Adult
Thompson et al. (2000)	Female	178	30.0	North America	High	African American	Low	Adult
Young et al. (2006)	Male	41,482	16.9	North America	High	Caucasian	Moderate	Adult
Emotional neglect								
Ansara et al. (2005)	Female	200	3.5	North America	High	--	Moderate	Adult
Bensley et al. (2003)	Female	3,527	14.4	North America	High	Caucasian	High	Adult
Chapman et al. (2004)	Female	5,108	13.2	North America	High	Caucasian	Moderate	Adult
Chapman et al. (2004)	Male	4,352	11.0	North America	High	Caucasian	Moderate	Adult
Clemmons et al. (2003)	Female	112	33.9	North America	High	Hispanic American	Moderate	Child
Elliott (1997)	Mixed	505	25.0	North America	High	Caucasian	Moderate	Adult
Fergusson & Horwood (1998)	Female	515	40.0	Australia	High	--	--	Adult
Fergusson & Horwood (1998)	Male	504	40.0	Australia	High	--	--	Adult
Gagné et al. (2005)	Female	622	23.6	North America	High	--	Low	Child
Jirapramukpitak et al. (2005)	Female	199	8.0	Asia	Low	--	--	Adult
Jirapramukpitak et al. (2005)	Male	144	9.7	Asia	Low	--	--	Adult
Scher et al. (2004)	Female	618	5.3	North America	High	Caucasian	Moderate	Adult
Scher et al. (2004)	Male	349	4.9	North America	High	Caucasian	Moderate	Adult
Stephenson et al. (2006)	Mixed	1240	80.1	Asia	Low	--	Low	Child
Thompson et al. (2000)	Female	178	33.0	North America	High	African American	Low	Adult
Young et al. (2006)	Male	41,482	15.4	North America	High	Caucasian	Moderate	Adult

[1]high means high-resource, low means low-resource; [2]for the subset of studies originating from North America; [3] -- means 'not reported'
